# Risk-adjusted benchmarking of long-term overall survival in patients with HER2-positive early-stage Breast cancer: A Swedish retrospective cohort study

**DOI:** 10.1016/j.breast.2023.05.008

**Published:** 2023-06-01

**Authors:** Jacob Thurell, Narges Manouchehri, Irma Fredriksson, Ulla Wilking, Jonas Bergh, Lisa Ryden, Linetta B. Koppert, Maria M. Karsten, Narsis A. Kiani, Elham Hedayati

**Affiliations:** a Department of Oncology-Pathology, Karolinska Institutet, Stockholm, Sweden; b Breast Cancer Center, Department of Breast, Endocrine Tumours and Sarcoma, Karolinska Comprehensive Cancer Center, Karolinska University Hospital, Stockholm, Sweden; c Department of Molecular Medicine and Surgery, Karolinska Institutet, Stockholm, Sweden; d Department of Clinical Sciences Lund, Division of Surgery, Lund University, Lund, Sweden; e Department of Surgery, Skane University Hospital, Malmö, Sweden; f Erasmus MC Cancer Institute, Dept of Surgery, Rotterdam, the Netherlands; g Charité – Universitätsmedizin Berlin, Department of Gynecology with Breast Center, Berlin, Germany; h Algorithmic Dynamics Lab, Center of Molecular Medicine, Karolinska Institutet, Stockholm, Sweden

**Keywords:** Survival analysis, Survival rate, Risk factor, Breast neoplasm, Female, Databases, Factual/statistics and numerical data, Quality improvement

## Abstract

**Aim:**

The main objective of the current study was to explore the value of risk-adjustment when comparing (i.e. benchmarking) long-term overall survival (OS) in breast cancer (BC) between Swedish regions. We performed risk-adjusted benchmarking of 5- and 10-year OS after HER2-positive early BC diagnosis between Sweden's two largest healthcare regions, constituting approximately a third of the total population in Sweden.

**Methods:**

All patients diagnosed with HER2-positive early-stage BC between 01-01–2009 and 31-12-2016 in healthcare regions Stockholm-Gotland and Skane were included in the study. Cox proportional hazards model was used for risk-adjustment. Unadjusted (i.e. crude) and adjusted 5- and 10-year OS was benchmarked between the two regions.

**Results:**

The crude 5-year OS was 90.3% in the Stockholm-Gotland region and 87.8% in the Skane region. The crude 10-year OS was 81.7% in the Stockholm-Gotland region and 77.3% in the Skane region. However, when adjusted for age, menopausal status and tumour biology, there was no significant OS disparity between the regions, neither at the 5-year nor 10-year follow-up.

**Conclusion:**

This study showed that risk-adjustment is relevant when benchmarking OS in BC, even when comparing regions from the same country that share the same national treatment guidelines. This is, to our knowledge, the first published risk-adjusted benchmarking of OS in HER2-positive BC.

## Highlights

• Risk-adjustment is crucial in accurately assessing and comparing breast cancer survival rates across different regions.
• A crude survival disparity favouring the Stockholm region was noted at 5 years of follow-up.
• The survival disparity disappeared at all time points after risk-adjustment for age, menopausal status and tumour biology.
• Future studies should assess if comorbidity burden and socioeconomic status could improve model accuracy.

## 1 Introduction

Benchmarking of health outcomes is an established method in Swedish breast cancer (BC) care [1] for identifying differences in performance between hospitals [2], and complements the Swedish national guidelines for BC care [3] to ensure equitable and high-quality care. Benchmarking entails collecting data on performance indicators from healthcare providers and comparing the results between the participating healthcare providers. Benchmarking BC outcome indicators (e.g. survival) is valuable but complex, as outcome variations can be caused by confounding factors outside the control of the healthcare provider [4], such as age and tumour biology. Adjusting results for the effect of these confounders (i.e. risk-adjustment) has been shown to improve the accuracy of benchmarking health outcomes outside the BC setting [5]. The National Quality Register for Breast Cancer (NKBC) of Sweden, launched in 2008, has a high coverage for quality assurance, benchmarking, and research [6]. It collects data in a shared national database and encompasses all BC cases' diagnostic and therapeutic processes and outcomes. The NKBC is tasked with performing annual national benchmarking of BC care.

BC is the most common cancer among women in Sweden and worldwide [7]. Both clinical demographic and tumour-related factors impact survival after BC diagnosis [3]. Human Epidermal Growth Factor Receptor-2 (HER2) is amplified in 14% of BC[8], a characteristic of tumour aggressiveness and poor prognosis [9]. The systemic treatment option for early-stage HER2-positive BC is adjuvant or neoadjuvant treatment with chemotherapy and one year of HER2-directed antibody treatment [3,10]. HER2-targeted therapy (HER2-therapy) has decreased mortality for HER2-positive BC by a third in early-stage HER2-positive BC [[11], [12], [13], [14]].

The 2021 annual report from the NKBC showed that 10-year OS for early-stage HER2-positive BC varied from 69% to 81% between the Swedish healthcare regions [1]. This could be a clinically relevant survival difference. Still, as the NKBC does not adjust their results for confounders like demographics and tumour characteristics, it is unclear whether survival differences are due to inter-regional differences in healthcare performance. Risk-adjustment might improve the accuracy and meaningfulness of BC survival comparisons. However, to our knowledge, no studies have assessed the value of risk-adjustment in benchmarking BC survival.

Our primary aim was therefore to assess the importance of risk-adjustment by comparing crude and adjusted 5-year survival rates and 10-year survival rates separately for early-stage HER2-positive BC using variables available in the NKBC. Secondly, we aimed to adjust for demographic and tumour-related factors and benchmark long-term survival rates in HER2-positive early-stage breast cancer patients between Stockholm-Gotland and Skane, Sweden's two largest healthcare regions.

## 2 Patients and methods

This study was approved by the Regional Ethics Review Board at the Karolinska Institute (Dnr 2012/745–31/1) and complied with the Declaration of Helsinki. As a result of Swedish legislation, patients included in national quality registers do not need to provide written informed consent for their data to be included in healthcare research (opt-out). The study protocol is listed on the ISRCTN registry (ISRCTN69229101) [15]. Results were reported according to the STROBE-criteria for cohort studies.

### 2.1 Data source

This was a retrospective register-based cohort study where information on participants where based on crossmatched data from the NKBC and the Swedish Cause of Death Register [16]. The Swedish National Board of Health and Welfare maintains the latter. Data from the cohort was linked by unique Personal Identification Numbers [17].

Diagnostic and treatment data for patients with early-stage HER2-positive BC in Stockholm‐Gotland or Skane Healthcare Regions of Sweden were obtained from the NKBC (Table 1). Data on the time of death was collected from the Swedish Cause of Death Register.

**Table 1:** Demographic and clinical characteristics of the patients in the two cohorts Stockholm-Gotland and Skane Healthcare Regions. Statistical testing for differences between the two cohorts is presented in the right column.

*Characteristics*	Stockholm-Gotland N (%)	Skane N (%)	P-value
**Total**	1631	1023	
**Age at BC diagnosis,***years*			
< 46	320 (19.6)	190 (18.6)	0.10
46–64	812 (49.8)	479 (46.8)	
≥65	499 (30.6)	354 (34.6)	
**Menopausal status**			
Premenopausal	569 (35.4)	316 (31.4)	**0.037**
Postmenopausal	1039 (64.6)	690 (68.6)	
Not given	21	17	
**ER-status**			
Positive	1060 (65.8)	667 (66.8)	0.60
Negative	550 (34.2)	331 (33.2)	
Not given	21	25	
**PR-status**			
Positive	734 (46.1)	442 (44.5)	0.42
Negative	858 (53.9)	552 (55.5)	
Not given	39	29	
**Histological grade**			
1	49 (3.2)	22 (2.4)	0.067
2	496 (32.4)	262 (28.7)	
3	988 (64.4)	629 (68.9)	
Not given	98	130	
**T-stage**			
0	11 (0.7)	5 (0.5)	**<0.001**
1	757 (46.7)	529 (52.4)	
2	662 (40.8)	408 (40.4)	
3	167 (10.3)	52 (5.1)	
4	24 (1.5)	16 (1.6)	
Not given	10	13	
**N-stage**			
0	953 (59.1)	535 (54.9)	**<0.001**
1	541 (33.6)	297 (30.5)	
2	78 (4.8)	90 (9.2)	
3	40 (2.5)	52 (5.3)	
Not given	19	49	
**Chemotherapy**^a^			
Yes	1172 (87.1)	704 (82.3)	**<0.001**
No	174 (12.9)	151 (17.7)	
Not given	54	23	
**Endocrine therapy**^a^			
Yes	841 (94.8)	526 (94.8)	1.00
No	46 (5.2)	29 (5.2)	
Not given	53	22	
**Radiation therapy**^a^			
Yes	1064 (80.9)	570 (66.6)	**<0.001**
No	251 (19.1)	286 (33.4)	
Not given	54	22	
**HER2-therapy**^a^			
Yes	1107 (84.1)	690 (80.6)	**0.016**
No	209 (15.9)	166 (19.4)	
Not given	54	22	

a For treatment variables, only data from September 2009 to the end of 2016 were available in the Stockholm region. For fair comparison, only patients diagnosed from 2006 to 09-01 to 2016-12-31 were included in the treatment data. Endocrine treatment is presented as the fraction of patients that had an indication for endocrine treatment (i.e. ER-positive patients) that also received such treatment. Data on the date of BC diagnosis, age at the time of BC diagnosis, tumour staging, nodal status, tumour biology (histological grade, estrogen receptor (ER) and progesterone receptor (PR) status), and received neo-adjuvant/adjuvant oncological treatment (chemotherapy, HER2-targeted therapy, endocrine therapy, and radiotherapy) were retrieved from the NKBC. Data on the time of death was collected from the Swedish Cause of Death Register.

### 2.2 Study population and cohorts

Women in the Stockholm‐Gotland and Skane Healthcare Regions (combined approximately 3.6 million inhabitants) above 18 at diagnosis of early-stage HER2-positive BC were included in the study. All patients were diagnosed between January 2008 and December 2016 and identified through NKBC. Dates of inclusion were chosen so that all patients had at least 5 years of follow-up, deemed the minimal follow-up time for clinical relevance. Start date of inclusion (2008-01-01) was chosen as it was the first year with adequate coverage in the NKBC. HER2-positivity was confirmed either with a result of 3+ on immunohistochemistry (IHC) or with a positive ISH-test (i.e. gene amplification) if the tumour scored as equivocal (IHC 2+).

Women with non-HER2-positive BC, primary metastatic (de novo) BC (defined as distant metastasis within three months from diagnosis date) and women without surgical resection of primary BC for other reasons were excluded.

Two study groups were formed: 1) Women with early-stage HER2-positive BC in the Stockholm-Gotland Healthcare Region (Stockholm-cohort); 2) Women with early-stage HER2-positive BC in the Skane Healthcare Region (Skane-cohort).

### 2.3 Covariates

We categorised age as ≤45 years, 46–64, and ≥65 years; menopausal status as pre- or postmenopausal; ER/PR status as positive or negative and histological grade as 1,2 or 3. The TNM classification system was used for tumour and lymph node staging [18]. In neoadjuvant-treated patients, T and N-stage at diagnosis were based on diagnostic biopsy, radiology or clinical examination. Oncological treatment, including chemotherapy, HER2-therapy, endocrine therapy, and radiotherapy, was reported for descriptive purposes and not planned to be included in the risk-adjustment models. This decision was made as we did not want to adjust for effects that directly reflect healthcare performance and are within control of the participating hospitals.

## 3 Outcome measures

The primary outcome measure in the current study was the 5- and 10-year OS from BC diagnosis. We compared crude and adjusted 5-and 10-year OS between the Stockholm-cohort and Skane-cohort.

OS was defined as the time (in years) between BC diagnosis and the end of follow-up (2022-02-09) or death.

### 3.1 Statistical analyses

Univariable Cox proportional hazards regression model was initially applied to identify covariates associated with all-cause death after BC diagnosis. A threshold of P-value<0.10 was initially set for the inclusion of the covariate in the multivariable Cox regression. The model was then reduced using backward elimination with a threshold of P-value<0.05 to consider the covariate statistically significant and retain it in the model. In model 1, we included patient characteristics as independent predictors. In model 2, we further added tumour biological characteristics to the model. Finally, model 3 was developed by reducing the statistically insignificant variables (threshold of P < 0.05 for retainment) from model 2 using backward elimination. Separate risk-adjustment models were developed for 5- and 10-year OS. Hazard ratios (HRs) with 95% confidence intervals were reported for each included covariate in the model. Our analysis accounts for the existence of two distinct cohorts from separate regions, and as such, we conducted two separate sets of Cox models. The first set encompasses patients with a minimum follow-up of 5 years, while the second set focuses on patients with a minimum follow-up of 10 years. We have reported both the crude survival estimates using the Kaplan-Meier method and the adjusted hazard ratios using the Cox proportional hazards model. The adjusted survival curves presented in the figures were estimated based on the Cox model with covariates set at their mean values. Survival disparities were statistically tested by applying the non-parametric log-rank test. Descriptive statistics and crude survival for the patients receiving HER2-therapy versus those not receiving HER2-therapy were reported (Supplementary Tables 1 and 2). The statistical software RStudio (4.2.2) and SPSS (V26) were used.

## 4 Results

**Fig. 1:**
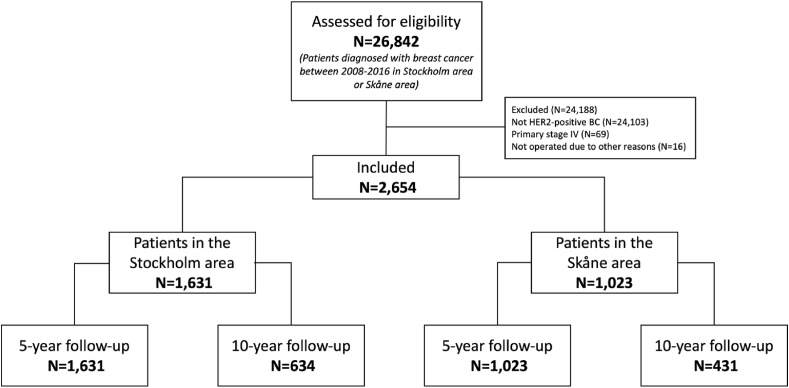
Flowchart of included patients.

26,842 patients were diagnosed with BC in the two Healthcare Regions between 2008 and 2016. Of these, 24,188 (90.1%) were excluded: 24,103 (89.8%) non-Her2 positive BC, 69 (0.3%) primary metastatic (de novo) BC and 16 (0.1%) without surgical resection of the primary BC, leaving a total of 2654 women that comprised the study population. Of these, 1631 (61.5%) patients belonged to the Stockholm-cohort and 1023 patients (38.5%) to the Skane cohort (Fig. 1).

Patient characteristics, tumour characteristics and treatments were summarised in Table 1. There was no significant difference in age distribution between the two cohorts. However, a higher proportion of the patients were postmenopausal in the Skane-cohort compared to the Stockholm-cohort (67.4% vs 63.7%; P = 0.037). The Stockholm-cohort had significantly higher tumour stage (P < 0.001) while the Skane cohort had higher nodal staging (P < 0.001). A higher fraction of the patients in the Stockholm-cohort received HER2-targeted therapies (84.1% vs 80.0%; P = 0.016) (Table 1). Median follow-up time using the reverse Kaplan-Meier could not be estimated as the probability does not drop to 0.5, which leads to an undefined follow-up time. Combined (for the two cohorts) median survival time was 100 months for the endpoint 5-year OS and 135 months for the enpoint 10-year OS. Around 99.0% of the patients that received HER2-targeted therapy also received chemotherapy in both cohorts (Supplementary Table 1). The Stockholm-Gotland cohort had a higher fraction of patients 65 years and older that received chemotherapy (74.4% vs 66.6%) and HER2-targeted therapies (74.4% vs 64.8%). Patients aged 65 and older received less chemotherapy and HER2-targeted therapies than the two younger subroups in both cohorts **(**Supplementary Table 3**)**.

The development of risk-adjustment models for 5- and 10-year OS are described in Table 2 and Table 3. ER/PR status did not pass the significant threshold in the univariable analyses and was not included in the multivariable analyses. Model 3, including a minimum number of statistically significant variables, was used as the final model at both 5- and 10-year OS. Postmenopausal status, tumour stage ≥2, and lymph node stage ≥2 were all strong predictors of worse survival at both 5- and 10-year OS. Age≥65 years strongly predicted worse survival at 10-year OS. The HRs, P-values and their CI for all 3 models are reported in Table 2.

**Table 2:** Development of 3 different risk-adjustment models for 5-year- overall survival among 2654 patients with HER2-positive early BC in the Stockholm-Gotland and Skane Healthcare Regions.

Model 1					Model 2					Model 3				
Covariates	Sig.	HR	95% CI for HR		Covariates	Sig.	HR	95% CI for HR		Covariates	Sig.	HR	95% CI for HR	
Age, years					Age, years					Age, years				
18-45*					18-45*					18-45*				
46–64	0.139	0.716	0.460	1.115	46–64	0.107	0.693	0.358	1.341	46–64	0.182	0.732	0.464	1.157
≥65	0.024	1.667	1.071	2.594	≥65	0.102	1.658	0.857	3.208	≥65	0.053	1.577	0.994	2.500
**Menopausal status**					**Menopausal status**					**Menopausal status**				
Premenopausal*					Premenopausal*					Premenopausal*				
Postmenopausal	<0.001	2.646	1.801	3.888	Postmenopausal	<0.001	2.535	1.418	4.533	Postmenopausal	**<0.001**	**2.559**	**1.714**	**3.820**
					**T-stage**					**T-stage**				
					0, 1*					0, 1*				
					2	<0.001	2.255	1.471	3.458	2	**<0.001**	**1.858**	**1.381**	**2.501**
					3, 4	<0.001	2.881	1.610	5.156	3, 4	**<0.001**	**3.549**	**2.542**	**4.956**
					**N-stage**					**N-stage**				
					0 *					0 *				
					1	<0.001	2.193	1.426	3.373	1	**<0.001**	**1.981**	**1.475**	**2.659**
					2,3	<0.001	4.052	2.550	6.439	2,3	**<0.001**	**2.420**	**1.612**	**3.632**
					**Histological grade**									
					1*									
					2	0.350	0.564	0.139	2.291					
					3	0.018	0.634	0.409	0.980					

**Abbreviations:** Significant (Sig), Hazard ratio (HR), Confident Interval (CI), Tumour-stage (T-stage), Nodal-stage (N-stage).

‘Reference Group.

**Table 3:** Development of 3 different risk-adjustment models for 10-year overall survival (OS) among 1065 patients with HER2-positive early BC in the Stockholm-Gotland and Skane Healthcare Regions.

Model 1					Model 2					Model 3				
Covariates	Sig.	HR	95% CI for HR		Covariates	Sig.	HR	95% CI for HR		Covariates	Sig.	HR	95% CI for HR	
Age, years					Age, years					Age, years				
18-45*					18-45*					18-45*				
46–64	0.378	0.750	0.454	1.239	46–64	0.455	0.821	0.490	1.377	46–64	0.394	0.803	0.486	1.329
≥65	0.026	1.616	0.964	2.708	≥65	0.013	1.972	1.153	3.373	≥65	**0.031**	**1.777**	**1.056**	**2.992**
**Menopausal status**					**Menopausal status**					**Menopausal status**				
Premenopausal*					Premenopausal*					Premenopausal*				
Postmenopausal	<0.001	2.333	1.518	3.586	Postmenopausal	0.000	2.322	1.495	3.606	Postmenopausal	**0.000**	**2.458**	**1.596**	**3.786**
					**T-stage**					**T-stage**				
					0 *					0 *				
					1	0.003	1.660	1.192	2.311	1	**0.009**	**1.545**	**1.114**	**2.143**
					2,3	<0.001	3.157	1.966	5.069	2,3	**0.001**	**1.978**	**1.341**	**2.917**
					**N-stage**					**N-stage**				
					0 *					0 *				
					1	0.004	1.632	1.168	2.280	1	**0.004**	**1.593**	**1.157**	**2.194**
					2,3	<0.001	2.120	1.427	3.150	2,3	**<0.001**	**2.746**	**1.748**	**4.313**
					**Histological grade**									
					1*									
					2	0.634	1.206	0.557	2.614					
					3	0.678	0.932	0.666	1.302					

**Abbreviations:** Significant (Sig), Hazard ratio (HR), Confident Interval (CI), Tumour-stage (T-stage), Nodal-stage (N-stage).

‘Reference Group.

### 4.1 Crude- and adjusted survival between stockholm and skane-cohort

**Fig. 2:**
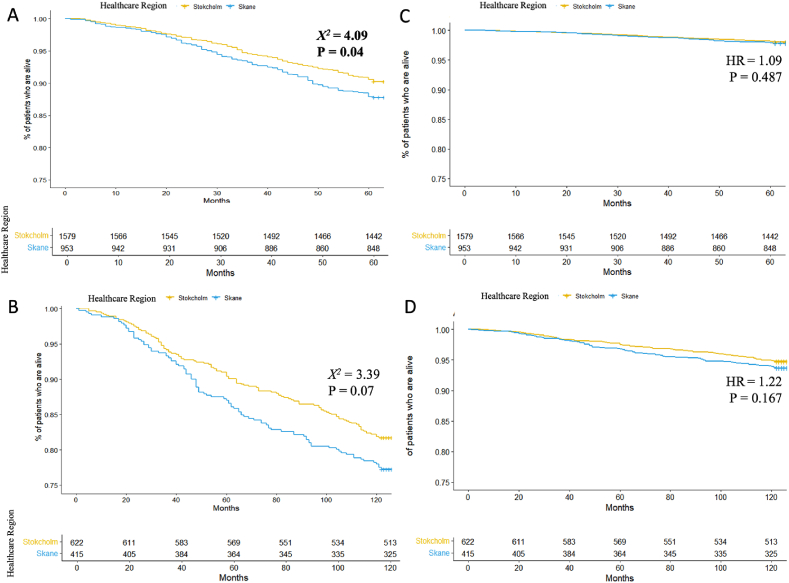
Kaplan-Meier curves (**A** and **B**) and adjusted Cox proportional hazards survival curves (**C** and **D**) demonstrating the benchmarking of 5- and 10-year overall survival (OS) in a total of 2654 female patients with HER2-positive early-stage BC (Stockholm-Gotland (n = 1631) and Skane (n = 1023) Healthcare Regions, Sweden) diagnosed 2008 to 2016. OS was defined as the time (in years) between BC diagnosis and the end of follow-up (February 9, 2022) or death. The Kaplan-Meier curves in **A** represent the crude 5-year OS (n = 2654) showing a significantly longer 5-year OS for patients in the Stockholm-cohort compared to Skane-cohort (Log-rank test: P = 0.04, chi-square (χ^2^) 4.09). The curves in **B** represent the crude 10-year OS (n = 1065). The adjusted Cox proportional hazards survival curves in **C** and **D** represent the adjusted 5- and 10-year OS in 2654 and 1065 patients, respectively. Hazard ratios (HRs) describe the hazard ratio of being in the Stockholm-Gotland cohort on 5- and 10-year adjusted OS. To enhance resolution and enable clearer differentiation and comparison, the survival curves have been truncated while maintaining the integrity of the data.

The crude 5-year OS was higher in the Stockholm-cohort than the Skane-cohort (90.3% vs 87.8%, P = 0.04, Fig. 2A), but the difference was not significant for crude 10-year OS (81.7% vs 77.3%, P = 0.07, Fig. 2B). When adjusted for confounders, there was no 5-year OS disparity between the cohorts (Fig. 2C). There was a trend of higher adjusted 10-year OS in the Stockholm cohort compared to the Skane cohort but this trend did not reach statistical significance (82.5% vs 78.3%, HR 1.22, P = 0.167) (Fig. 2D). In subgroup analyses, patients treated with HER2-treatment had higher 5- and 10-year OS than the patients that did not receive such treatment in both cohorts (Supplementary Table 2).

## 5 Discussion

Performing risk-adjustment when benchmarking BC survival improved accuracy. The crude 5-year OS disparity (90.3% vs 87.8%, P = 0.04) did not remain statistically significant after adjustment for the patient- and tumour characteristics (91.3% vs 89.0%, HR 1.09, P = 0.487). The crude 10-year OS was 81.7% in Stockholm‐Gotland region and 77.3% in the Skane healthcare region, higher than the national average among all Swedish healthcare regions of 74% [1].

Tumour- and lymph node stages ≥2 had a negative impact on both 5- and 10-year OS. These findings were expected as they are well-established risk factors of death after BC diagnosis in all BC subtypes [18]. Patients above 65 have a worse 10-year OS. The main cause of this is most likely that elderly patients have a lower expected 10-year OS in general than younger patients. This effect might have been enhanced by the increased risk of severe chemotherapy-induced toxicity in elderly patients. However, no standard treatment exists for these patients; thus, in clinical practice, they are often given lower doses of chemotherapy or single HER2-therapy, with a lower efficacy [19]. This is also in line with our results, as we can show that patients above 65 years received less chemotherapy and anti-HER2 therapy than the younger patient groups. Furthermore, being postmenopausal was a robust independent predictor of death at both time points, even when adjusting for age. Our findings emphasise the importance of including TN-stage and menopausal status when developing risk-adjustment models for BC survival.

Despite national guidelines [3], there are regional differences in how BC is treated in Sweden. In our study, the proportion of patients that received HER2-therapy was 84.1% in the Stockholm‐Gotland region and 80.6% in the Skane region. A higher fraction of the patients in the Stockholm-Gotland cohort also received chemotherapy. This was surprising, as the patients in the Skane cohort generally had more aggressive tumours with higher N-stage, indicating that more patients in the Skane cohort would benefit from more aggressive BC treatment. However, these potential deviations from national guidelines could be adequate as the Skane cohort also had older patients that might have been deemed unfit for chemotherapy and HER2-therapy.

To our knowledge, no other studies have performed risk-adjustment when benchmarking survival in HER2-positive early-stage BC between national regions or countries. The most relevant comparisons are studies evaluating the real-world effectiveness of adjuvant trastuzumab [20,21]. The real-world RETROHER-study [20] followed 573 patients that had received adjuvant trastuzumab treatment from 10 Italian hospitals. They reported 93% 10-year OS among patients that had received trastuzumab which is higher than the 86% 10-year OS in both regions in the current study. An Australian whole-population study [21] investigated 14,644 patients with HER2-positive BC that received adjuvant trastuzumab. They reported a 9-year observed OS of 90%, which is in line with our findings. Risk-adjustment would, however, be required to assess survival differences between these international cohorts accurately.

A study by Vos et al. [22] showed that risk-adjustment impacted BC's surgical outcomes and process indicators. Even though they did not assess survival outcomes, their results complements ours in emphasizing the importance of risk-adjustment when benchmarking different indicators in BC. There are currently no validated risk-adjusted models for BC survival outcomes. This is likely why neither NKBC's annual benchmarking of BC survival [1] nor the published international comparisons of BC [23,24] have risk-adjusted their results. The EUROCARE-5 [23] and the CONCORD-3 [24] publish large-scale un-adjusted benchmarking of cancer-specific survival between several countries, including Sweden. Risk-adjustment would probably improve meaningfulness in such international benchmarking of BC survival as variations in genetic predisposition, socioeconomy, and demographics can be assumed to be larger between countries than within a small country like Sweden.

We plan to validate the developed risk-adjustment model and perform risk-adjusted benchmarking, including all Swedish regions. When validated, our risk-adjustment models could be applied in the annual national benchmarking of BC care in NKBC. Risk-adjustment models can contribute to a better basis for identifying inequalities in BC care at the hospital/region/country level that can be used in improvement projects and quality development. Moreover, it is of the essence for a small country like Sweden to compare outcomes with larger comparable countries.

Overall, this study highlights the importance of risk-adjustment when benchmarking survival in patients with HER2-positive early-stage BC and suggests that differences in patient composition regarding clinical demographic and tumour-related factors may explain observed survival disparities between different regions. The study also identifies important predictors of survival, including TN-stage and menopausal status, and provides insights into potential reasons for survival disparities.

Strengths of this study include the use of data from high-quality Swedish registers, which contain few dropouts or data gaps, suggesting that the internal validity of this study is substantial [6]. We have used a rigorous multi-step risk-adjustment model and included the minimum required number of variables that pass the significance test in the model. This approach increases the statistical power and generalisability of the result. Future research can build on these findings to identify and address inequalities in the standard of care and extend the follow-up length to assess disease-free survival. This study has several limitations. It is important to note that the current study focused specifically on HER2-positive early-stage BC, and the results may not be generalisable to other subtypes of BC. Is also important to emphasise that the developed risk-adjustment lack certain variables that could impact survival (i.e. residual confounding). First, it would have been valuable to include the prognostic factor KI-67. Unfortunately the NKBC only had data available on this variable from the start of 2013, why we chose to exclude it from the risk-adjustment models. Furthermore, the inclusion of comorbidity burden, ECOG performance status at diagnosis and socioeconomic factors could also have improved the accuracy of the risk-adjustment models. These factors were not available in the NKBC. While the inclusion of these factors probably would improve model accuracy, we do not believe that these additions would change our conclusion that risk-adjustment is important when benchmarking HER2-positive early BC survival. Our primary objective in this study was to emphasise the significance of risk-adjustment in survival analysis, even when considering a minimal number of variables. We therefore opted to focus on showcasing the importance of risk-adjustment itself rather than comparing different methods with a broader set of covariates. In variable selection, we consider which variables are expected to exhibit substantial regional differences, and its influence is not reflected in other variables. Future studies should continue optimising risk-adjustment models by testing whether inclusion of comorbidity burden, socioeconomic variables and ECOG performance status improve the predictive capability of the models. Our study does not provide information about treatment data for the Stockholm-cohort from 2008 to 01-01 to 2009-09-30. This does not affect the risk-adjusted survival as we do not adjust the results for treatments but limits us from drawing any conclusions regarding how treatment differences might have impacted survival disparities. Finally, the study could only assess overall survival due to data availability. Including breast cancer specific survival as an outcome could have further illuminated breast cancer specific survival disparities.

## 6 Conclusions

In summary, the study demonstrates the feasibility and importance of risk-adjusted benchmarking for HER2-positive early-stage BC in Sweden using readily available data from a quality register. The study found significant crude survival disparities between the Stockholm-Gotland and Skane regions, which were no longer significant after adjusting for relevant confounders. These findings support the hypothesis that risk-adjustment can enhance the accuracy and quality of benchmarking in BC care. We plan to refine and validate the risk-adjustment model to be applicable in all Swedish regions. We plan to perform national and international risk-adjusted benchmarking of BC survival.

## Ethics approval

This study complied with the Declaration of Helsinki, and it was approved by the Regional Ethics Review Board at Karolinska Institutet (Dnr: 2012/745–31/1). Permission was obtained to access and use the national registries and review the medical records.

## Funding

This work was supported by a grant from the Swedish Breast Cancer Association (BCF). We also acknowledge the support of the 10.13039/501100000038Natural Sciences and Engineering Research Council of Canada (NSERC), [funding reference number PDF568028-2022]. Cette recherche a été financée par le Conseil de recherches en sciences naturelles et en génie du Canada (10.13039/501100000038CRSNG), [numéro de référence PDF568028-2022].

## Author contributions

All authors contributed to the study's conception and design. JT, NM, UW, NK and EH performed material preparation, data collection, and analysis. JT wrote the manuscript's first draft, and all authors commented on subsequent versions. All authors read and approved the final manuscript.

## Consent to participate

Based on previous Swedish legislation, patients registered in national quality registries do not need to provide written informed consent for their data to be included in healthcare research; however, they are informed that their data is included in the registries and that they could be removed at any time.

## Consent to publish

Based on previous Swedish legislation, patients registered in the national quality registries do not need to provide written informed consent for their anomymized data to be included in healthcare research and/or to be published.

## Declaration of competing interest

Financial interests: **JB** has received research grants from Amgen, AstraZeneca, Bayer, Merck, Pfizer, Roche and Sanofi-Aventis to Karolinska Institutet and/or University Hospital. No personal payments; JB is co-author of a chapter on” Prognostic and Predictive factors in early, non-metastatic breast cancer” in UpToDate. Honoraria to Asklepios Medicin HB; JB has Stocks in Stratipath AB, being involved in AI-based diagnostics for breast cancer. JB is chairperson for Coronis and Asklepios Cancer Research AB. JB has received honoraria from Roche and AstraZeneca for chairmanship and lectures at scientific meetings and consultations for Stratipath AB.

**EH** receives lecture fees and/or research funding from 10.13039/100004337Roche AB, 10.13039/100002429Amgen AB, 10.13039/100004319Pfizer AB, and 10.13039/100013226Pierre Fabre, all paid to Karolinska University Hospital.

**IF** has received institutional research grants from MSD, unrelated to the current work.

**LR**has received honoraria from Onkologisk tidskrift, Denmark, for lecturing.

**NAK** has received funding from the 10.13039/501100000780European Union under the 10.13039/501100007601Horizon 2020 programme (MultipleMS grant agreement 733,161. All remaining authors declare that they have no conflict of interest.
