# Supplementary material for: Risk-adjusted benchmarking of long-term overall survival in patients with HER2-positive early-stage Breast cancer: A Swedish retrospective cohort study
Source: Breast. 2023 Jun 1;70:18–24. doi: 10.1016/j.breast.2023.05.008 (PMC10272493; doi:10.1016/j.breast.2023.05.008)
Supplement: Multimedia component 1 [file mmc1.docx]

**Supplements**

| **Supplementary Table 1.** Descriptive statistics of the subgroups that received HER2-targeted therapy versus no HER2-targeted therapy versus the patients with missing data on HER2 treatment in the Stockholm-Gotland and Skane regions. | | | | | | |
| --- | --- | --- | --- | --- | --- | --- |
| ***Characteristics*** | **Stockholm-Gotland**  N=1,631  n (%) | | | **Skane**  N=1,023  n (%) | | |
|  | **No HER2-targeted therapy** | **HER2-targeted therapy** | **Missing HER2-therapy data^†^** | **No HER2-targeted therapy** | **HER2-targeted therapy** | **Missing HER2-therapy data**^†^ |
| **Total** | 215 | 1138 | 278 | 194 | 773 | 56 |
| **Age at BC diagnosis,** *years* |  |  |  |  |  |  |
| < 46 | 27 (12.5) | 226 (19.9) | 67 (24.1) | 26 (13.3) | 158 (20.4) | 6 (10.7) |
| 46–64 | 81 (37.7) | 600 (52.7) | 131(67.1) | 44 (22.7) | 405 (52.4) | 30 (53.6) |
| ≥ 65 | 107 (49.8) | 312 (27.4) | 80 (28.8) | 144 (64.0) | 210 (27.2) | 20 (35.7) |
| **Menopausal status** |  |  |  |  |  |  |
| Premenopausal | 28 (13.0) | 442 (38.9) | 99 (35.6) | 29 (15.0) | 272 (35.2) | 15 (26.8) |
| Postmenopausal | 183 (85.1) | 677 (59.5) | 179 (64.4) | 163 (84.0) | 487 (63.0) | 40 (71.4) |
| Not given | 4 (1.9) | 19 (1.6) | 0 (0.0) | 2 (1.0) | 14 (1.8) | 1 (1.8) |
| **ER-status** |  |  |  |  |  |  |
| Positive | 155 (72.1) | 732 (64.3) | 173 (62.2) | 133 (68.6) | 497 (64.3) | 37 (66.1) |
| Negative | 58 (27.0) | 390 (34.3) | 102 (36.7) | 58 (29.9) | 255 (33.0) | 18 (32.1) |
| Not given | 2 (0.9) | 16 (1.4) | 3 (1.1) | 3 (1.5) | 21 (2.7) | 1 (1.8) |
| **PR-status** |  |  |  |  |  |  |
| Positive | 112 (52.1) | 515 (45.3) | 107 (38.5) | 88 (45.4) | 326 (42.2) | 28 (50.0) |
| Negative | 102 (47.4) | 590 (41.8) | 166 (59.7) | 103 (53.1) | 422 (54.6) | 27 (48.2) |
| Not given | 1 (0.5) | 33 (2.9) | 5 (1.8) | 3 (1.5) | 25 (3.2) | 1 (1.8) |
| **Histological grade** |  |  |  |  |  |  |
| 1 | 11 (5.1) | 26 (2.3) | 12 (4.3) | 8 (4.1) | 13 (1.7) | 1 (1.8) |
| 2 | 78 (36.3) | 344 (30.2) | 74 (26.6) | 66 (34.0) | 180 (23.3) | 16 (28.6) |
| 3 | 117 (54.4) | 698 (61.3) | 173 (62.3) | 110 (56.7) | 467 (60.4) | 32 (57.1) |
| Not given | 9 (4.2) | 70 (6.2) | 19 (6.8) | 10 (5.2) | 113 (14.6) | 7 (12.5) |
| **T-stage** |  |  |  |  |  |  |
| 0 | 0 (0.0) | 11 (1.0) | 0 (0.0) | 2 (1.0) | 2 (0.3) | 1(1.8) |
| 1 | 113 (52.6) | 502 (44.1) | 142 (51.1) | 103 (53.1) | 401 (51.9) | 25 (44.6) |
| 2 | 82 (38.1) | 473 (41.6) | 107 (38.5) | 76 (39.2) | 310 (40.1) | 2 (39.3) |
| 3 | 17 (7.9) | 129 (11.3) | 21 (7.6) | 9 (4.6) | 38 (4.9) | 5 (8.9) |
| 4 | 1 (0.5) | 17 (1.5) | 6 (2,2) | 0 (0.0) | 13 (1.7) | 3 (5,4) |
| Not given | 2 (0.9) | 6 (0.5) | 2 (0.6) | 4 (2.1) | 9 (1.1) | 0 (0.0) |
| **N-stage** |  |  |  |  |  |  |
| 0 | 136 (63.3) | 658 (57.8) | 159 (57.2) | 110 (56.7) | 404 (52.3) | 21 (37.5) |
| 1 | 49 (22.8) | 395 (34.7) | 97 (34.9) | 44 (22.7) | 237 (30.7) | 16 (28.6) |
| 2 | 8 (3.7) | 53 (4.7) | 17 (6.1) | 15 (7.7) | 65 (8.4) | 10 (17.8) |
| 3 | 8 (3.7) | 27 (2.4) | 5 (1.8) | 9 (4.6) | 36 (4.6) | 7 (12.5) |
| Not given | 14 (6.5) | 5 (0.4) | 0 (0.0) | 16 (8.3) | 31 (4.0) | 2 (3.6) |
| **Chemotherapy**^†^ |  |  |  |  |  |  |
| Yes | 51 (23.7) | 1123 (98.7) | 0 (0.0) | 21 (11.0) | 766 (99.1) | 1 (1.8) |
| No | 164 (76.3) | 15 (1.3) | 0 (0.0) | 171 (88.0) | 7 (0.9) | 0 (0.0) |
| Not given |  |  | 278 (100) | 2 (1.0) |  | 1 (98.2) |
| **Endocrine therapy**^†^ |  |  |  |  |  |  |
| Yes | 143 (66.5) | 398 (65.0) | 0 (0.0) | 111 (57.2) | 523 (67.7) | 0 (0.0) |
| No | 72 (33.5) | 740 (35.0) | 0 (0.0) | 83 (42.8) | 250 (32.3) | 0 (0.0) |
| Not given |  |  | 278 (100) |  |  | 56 (100) |
| **Radiation therapy**^†^ |  |  |  |  |  |  |
| Yes | 137 (63.7) | 552 (83.8) | 0 (0.0) | 79 (40.1) | 568 (73.5) | 0 (0.0) |
| No | 77 (35.8) | 275 (16.2) | 0 (0.0) | 115 (59.9) | 205 (16.5) | 0 (0.0) |
| Not given | 1 (0.5) | 0 (0.0) | 278 (100) | 0 (0.0) | 0 | 56 (100) |
| **HER2-therapy** |  |  |  |  |  |  |
| Yes | 0 (0.0) | 1138 (100) | 0 (0.0) | 0 (0.0) | 773 (100) | 0 (0.0) |
| No | 215 (100) | 0 (0) | (0.0) | 194 (100) | 0 (0) | 0 (0.0) |
| Not given | 0 (0.0) | 0 (0.0) | 278 (100) | 0 (0.0) | 0 (0.0) | 56 (100) |
| ^†^For treatment variables, only data from September 2009 to the end of 2016 were available in the Stockholm region. For fair comparison, only patients diagnosed from 2006-09-01 to 2016-12-31 were included in the treatment data. Endocrine treatment is presented as the fraction of patients that had an indication for endocrine treatment (i.e. ER-positive patients) that also received such treatment. Data on the date of BC diagnosis, age at the time of BC diagnosis, tumour staging, nodal status, tumour biology (histological grade, estrogen receptor (ER) and progesterone receptor (PR) status), and received neo-adjuvant/adjuvant oncological treatment (chemotherapy, HER2-targeted therapy, endocrine therapy, and radiotherapy) were retrieved from the NKBC. Data on the time of death was collected from the Swedish Cause of Death Register. | | | | | | |

| **Supplementary Table 2.** Crude (un-adjusted) 5-and 10-year overall survival (OS) expressed in percentages for the subgroups that received versus did not receive HER2-targeted therapy in each of the Stockholm-Gotland and Skane regions. (n) equals the number of patients included in the analyses at each time point. | | | | | | |
| --- | --- | --- | --- | --- | --- | --- |
| ***OS-year*** | **Stockholm-Gotland**  % (n) | | | **Skane**  % (n) | | |
|  | **All patients** | **No HER2-targeted therapy** | **HER2-targeted therapy** | **All patients** | **No HER2-targeted therapy** | **HER2-targeted therapy** |
| **5-year OS** | 90.3 (1631) | 75.3 (215) | 93.8 (1138) | 87.8 (1023) | 69.6 (194) | 93.7 (773) |
| **10-year OS** | 81.7 (634) | 61.8 (83) | 85.6 (326) | 77.3 (431) | 50.6 (83) | 85.8 (303) |

| **Supplementary Table 3.** Descritption of given treatment divided by age groups in both cohorts. | | | | | | |
| --- | --- | --- | --- | --- | --- | --- |
| ***Characteristics*** | **Stockholm-Gotland**  N=1,391  n (%) | | | **Skane**  N=878  n (%) | | |
|  | **<45 years** | **45-64 years** | **Above 65 years** | **<45 years** | **45-64 years** | **Above 65 years** |
| **Chemotherapy**^†^ |  |  |  |  |  |  |
| Yes | 232 (93.9) | 617 (91.7) | 311 (74.4) | 146 (89.6) | 357 (91.5) | 201 (66.6) |
| No | 15 (6.1) | 56 (8.3) | 107 (25.6) | 17 (10.4) | 33 (8.5) | 101 (33.4) |
| Not given | 9 | 22 | 22 | 0 | 9 | 14 |
| **Endocrine therapy**^†^ |  |  |  |  |  |  |
| Yes | 147 (93.0) | 428 (94.7) | 255 (96.2) | 106 (91.4) | 248 (96.5) | 172 (94.5) |
| No | 11 (7.0) | 24 (5.3) | 10 (3.8) | 10 (8.6) | 9 (3.5) | 10 (5.5) |
| Not given | 9 | 22 | 22 | 0 | 10 | 12 |
| **Radiation therapy**^†^ |  |  |  |  |  |  |
| Yes | 213 (86.6) | 561 (83.4) | 307 (73.4) | 109 (66.9) | 286 (73.5) | 175 (57.6) |
| No | 33 (13.4) | 112 (16.6) | 111 (26.6) | 54 (33.1) | 103 (26.5) | 129 (42.4) |
| Not given | 10 | 22 | 22 | 0 | 10 | 12 |
| **HER2-therapy**^†^ |  |  |  |  |  |  |
| Yes | 222 (89.9) | 592 (88.0) | 311 (74.4) | 139 (85.3) | 354 (91.0) | 197 (64.8) |
| No | 25 (10.1) | 81 (12.0) | 107 (25.6) | 24 (14.7) | 35 (9.0) | 107 (35.2) |
| Not given | 9 | 22 | 22 | 0 | 10 | 12 |
| ^††^For treatment variables, only data from September 2009 to the end of 2016 were available in the Stockholm region. For fair comparison, only patients diagnosed from 2006-09-01 to 2016-12-31 were included in the treatment data. Endocrine treatment is presented as the fraction of patients that had an indication for endocrine treatment (i.e. ER-positive patients) that also received such treatment. Data on the date of BC diagnosis, age at the time of BC diagnosis, tumour staging, nodal status, tumour biology (histological grade, estrogen receptor (ER) and progesterone receptor (PR) status), and received neo-adjuvant/adjuvant oncological treatment (chemotherapy, HER2-targeted therapy, endocrine therapy, and radiotherapy) were retrieved from the NKBC. Data on the time of death was collected from the Swedish Cause of Death Register. | | | | | | |
